# Evaluating the Thermal Conductivity of Hemp-Based Insulation

**DOI:** 10.3390/ma18081723

**Published:** 2025-04-09

**Authors:** Thomas Fiedler, James Pedersen

**Affiliations:** 1 School of Engineering, The University of Newcastle, Callaghan 2308, Australia; 2 School of Architecture, The University of Newcastle, Callaghan 2308, Australia; james.pedersen@newcastle.edu.au

**Keywords:** thermal conductivity, thermal insulation, hemp, sustainable building materials, low-carbon insulation

## Abstract

This study evaluates the thermal conductivity of hemp-based insulation materials, focusing on loose bulk mixtures of hemp fibre and hurd. Transient Plane Source (TPS) measurements were employed to assess the thermal conductivity of these materials, with a controlled variation in the fibre-to-hurd ratio and bulk density. Samples from various suppliers, including those with different fibre diameters and hurd contents, were tested. The results indicate thermal conductivities ranging from 0.055 to 0.065 W/mK, demonstrating good insulation performance. This study also highlights the influence of sample compression on thermal conductivity, with higher compression leading to both increased bulk density and thermal conductivity. When compared to the conventional insulation materials glass wool and polystyrene, hemp-based materials exhibited approximately double the thermal conductivity. However, the same thermal resistance (R-value) could be achieved by increasing the thickness of the hemp-based insulation.

## 1. Introduction

This thermal performance study set out to determine whether unmanufactured loose mixtures of hemp fibre and hemp hurd possess sufficiently low thermal conductivity such that they could be viably used as insulation in buildings. Building insulation works by slowing the passage of heat conducted through the building envelope and therefore reduces heat loss from a building interior in cold weather and heat gain to the interior in warm weather. Insulation also reduces the mechanical heating and cooling load to a building required to retain occupant comfort, and in turn reduces the operational carbon of the building, the carbon dioxide emissions associated with the mechanical heating and cooling of the building. For building insulation to be effective, it will typically fill the roofs, walls and floors around the building’s structural framing and can therefore constitute a significant proportion of a building’s volume and mass. As such, insulation also contributes significantly to the building’s embodied carbon, the carbon dioxide emissions associated with material manufacture and building construction. Careful specification of building insulation is therefore paramount since buildings are estimated to account for 32% of total global energy use [1] and 31% of global carbon dioxide emissions, 50% of which resulting from residential building operational energy use, 32% resulting from non-residential building operational energy use, and 18% associated with the embodied energy of buildings [2].

This study tested unmanufactured loose mixtures of hemp fibre and hurd to determine their efficacy in reducing operational carbon in buildings. The most extensive use of hemp in buildings is most commonly ‘hempcrete’ or hemp insulation batts. ‘Hempcrete’ is the mixture of hemp hurd (the woody inner portion of the hemp stalk) with lime mortar and water, with the mixture sprayed or cast in a method that is similar to forming concrete [3]. Hemp insulation batts are made by mixing hemp fibre (the bast fibre that surrounds the hemp stalk) with smaller proportions of adhesive fibres that bind the mixture when processed with heat [4]. In comparison, this study tests unmanufactured hemp mixtures for their potential to provide a lower-cost and less carbon-intensive alternative due to their comparatively unprocessed state. Previous research suggests that hemp fibre possesses sufficiently low thermal conductivity and high thermal resistance to be capable of serving as insulation in buildings. Kosiński et al. determined that the Białobrzeskie variety of hemp fibre is capable of achieving a thermal conductivity of 0.040 W/mK when tested at a temperature of 10 °C and a density of 85 kg/m^3^ and explained that an increase in density improves thermal resistance due to limiting the air flow through the test sample [5]. Stapulionienė et al. discovered a thermal conductivity of 0.0341 W/mK at an increased hemp fibre density of 102.5 kg/m^3^ [6]. Kremensas et al.’s studies tested at 10 °C and added polylactide (PLA) binders to their hemp fibre mix. When the PLA binder constituted 15% of the mix, the thermal conductivity result of 0.0367 W/mK was similar to that of other tests at similar high density (100 kg/m^3^); however, conductivity results at the lower test density of 40 kg/m^3^ produced a lower conductivity (more insulating) result of 0.0383 W/mK when compared to non-bound results such as Kosiński et al.’s 0.051 W/mK at 40 kg/m^3^ [4]. Sair et al. tested their fibre at a range of different temperatures and discovered that the thermal conductivity of hemp fibre was optimised at 0.04 W/mK when the testing environment was cooled to a minimum temperature of 10 °C [7]. Thermal conductivity of these levels is consistent with that of widely commercially available insulation products such as mineral wool [8]. Our study expands the field by comparing the thermal performance of a range of hemp samples by sourcing the mixes from three separate geographic locations demonstrating different levels of decortication and experimented with varying quantities of hemp hurd added to each mix.

There are broad ongoing efforts to refine insulation materials and methods in buildings and their components, with particular noteworthy advances in versatility and thermal resistance in applications such as nanofibre technology [9] and phase change materials [10,11,12]. This study chose hemp as its subject not so much on the basis of its capacity for thermal resistance but more on the basis of the material’s capacity to absorb significant amounts of atmospheric carbon during growth and therefore act to potentially mitigate the overall embodied carbon in buildings [13]. According to the Evah Institute, hemp fibre and hurd has an embodied carbon of negative 1.4 kg for each kilogram of hemp fibre, a figure which accounts for CO_2_ emissions associated with harvesting, decortication, transport, and storage offset by the CO_2_ sequestered during the growth of the hemp crop [14]. This means that for each kilogram of hemp used in construction, 1.4 kg of CO_2_ will be drawn from the atmosphere. Therefore, 4000 kg of a hemp fibre and hurd mix incorporated as insulation in a standard residential construction would result in 5600 kg of CO_2_ drawn from the atmosphere. Hence, if such a hemp fibre and hurd mix was used in standard Australian residential volume housing, which is typically built using treated pine framed brick veneer and concrete slab construction with aluminium door and window framing, then the CO_2_ drawdown associated with the hemp use would have the potential to offset over 13% of the house’s embodied carbon [15]. Further potential practical application of hemp mix insulation is in lighter and more biogenic-based construction, which therefore has potential for even greater carbon offset.

## 2. Methodology

### 2.1. Sample Preparation

**Figure 1:**
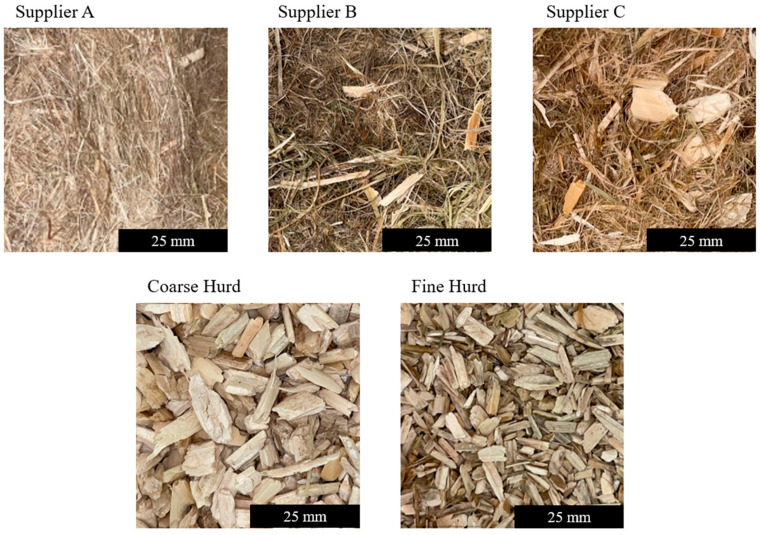
Bulk hemp mixtures.

Hemp mixtures were obtained in bulk from three different suppliers: one located in France and two in Australia. The samples were dried prior to testing. For analysis, samples were randomly subdivided into testing volumes of 9 L. This volume was selected to ensure a representative sample and sufficient thermal diffusion depth for Transient Plane Source analysis. Photographs of the hemp are shown in Figure 1. Supplier A provided fibres only, with a comparably smaller diameter. Supplier B provided fibres with a larger diameter and low hurd content. Supplier C offered fibres of similar size to those of Supplier B, but with a higher hurd content. Between measurements, samples were manually fluffed and mixed to create varied testing surfaces.

In addition to hemp fibre mixtures, additional hurd-only samples were considered for testing (see Figure 1, second row). Due to the limited availability of hurd compared to fibre, the testing volume for hurd-only samples was reduced to 4.5 L. In contrast to the fibre samples, the hurd did not compress significantly after loading, resulting in a similar TPS measurement volume to that of the fibre samples. Hurd particles were separated into two size categories: fine hurd with a particle size between 5 and 10 mm and coarse hurd with a particle size between 10 and 25 mm.

Following initial testing, the received material from Supplier B was manually separated into fibres and hurd. The resulting fibre-only sample was tested before a small amount of hurd was added to achieve a targeted uncompressed fibre volume fraction of 90%. The mixture was thoroughly blended to ensure uniform distribution before retesting. This process was repeated for fibre volume fractions of 75%, 50%, and 25%. Finally, a hurd-only sample was tested with a decreased testing volume of 4.5 L.

All samples were received in a dried state from the suppliers and stored in a climate-controlled room at 22 °C to 26 °C with a relative humidity of 40% to 50%. They were kept for at least three weeks before testing under the same environmental conditions.

### 2.2. Bulk Density Measurements

**Figure 2:**
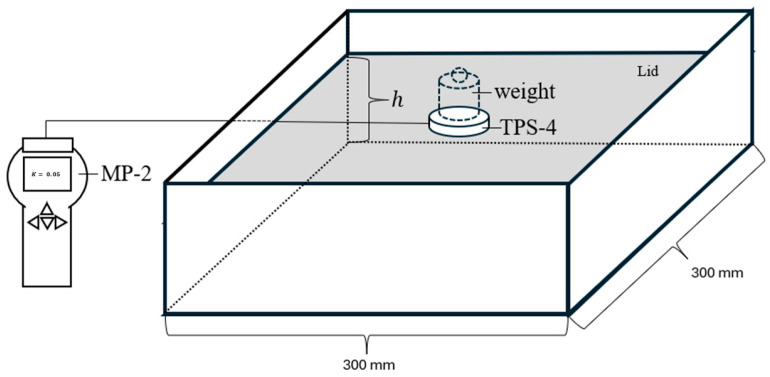
Enclosure for bulk density and thermal testing.

First, samples were separated from bulk materials by loosely filling a vessel to the target volume of either 4.5 (hurd-only) or 9 L (fibre mixtures). Next, a precision scale (accuracy ±0.1 g) was used to determine the mass *m* of each sample. The samples were then inserted into an enclosure box (see Figure 2) with floor dimensions of A = 300 mm × 300 mm. Material was added gradually in small batches to ensure uniformity. Based on the sample volume, for fibre mixtures, the initial fill height h in the enclosure box was 100 mm, whereas hurd samples had a corresponding fill height of 50 mm. To minimise thermal contact resistance during Transient Plane Source (TPS) measurements, a compression load of either 1 kg, 3 kg, or 5 kg was applied to the upper lid. The application of compression caused a significant reduction in fill height, particularly for fibre samples, which are more prone to compression. The bulk density for each sample and compression load was calculated using the relationship

$$\rho_{B}=\frac{m}{A\cdot h}$$

using the known floor area A=0.09 m^2^, alongside the measured mass m and compressed fill height h.

### 2.3. Transient Plane Source Measurements

The Transient Plane Source (TPS) measurements were conducted using the MP-2 system produced by Thermtest Instruments (Hanwell, NB, Canada), paired with the TPS-4 sensor from the same manufacturer. The single-sided sensor design adheres to conventional TPS theory [16] and, according to the manufacturer, maintains accuracy within a 5% error margin and repeatability within a 2% error [17].

The TPS technique relies on a sensor that acts both as a heat source and a temperature sensor. When a current is passed through the sensor, it generates heat, and the sensor’s resistance changes in response to the temperature increase. The temperature change over time is monitored, and this transient thermal response is used to calculate the thermal conductivity of the material. The TPS sensor acts as both a heat source and a temperature sensor. As heat dissipates into the material, the temperature rise ΔT(t) at the sensor is modelled based on transient heat conduction theory. The thermal conductivity k is determined by fitting the measured ΔT(t) curve to a theoretical model, using the known geometry (sensor radius 31.5 mm) and power input [11].

Prior to each testing sequence, the system was calibrated using a Pyrex reference sample of known thermal conductivity, k=1.15 K (at 300 K [18]), to ensure accurate measurements. In all cases, a deviation below 2.3% from the expected thermal conductivity was obtained. For insulation materials with thermal conductivity in the range of 0.03 to 0.5 W/mK, the low-power setting of 40 mA was selected. Each measurement lasted approximately 60 s, and each test was repeated five times for consistency. Between tests, the sample was manually mixed to alter the testing surface and then repacked using the selected compression weight.

The inherent heterogeneity of hemp fibres and their mixtures with hurd poses a challenge to achieving uniform thermal property measurements. The Transient Plane Source method assesses thermal properties in the immediate vicinity of the sensor–sample contact. To enhance measurement representativeness, a large sensor was selected, providing a contact area of 3117 mm^2^. This ensured coverage of a substantial sample cross-section, minimising variability. Samples were agitated between measurements, yet only minor deviations were observed, indicating consistency. The relatively large measurement volume supports the assumption that a representative volume element (RVE) was considered. Furthermore, a previous study [19] using alternative measurement techniques to characterise wool fibres—materials with a similar topology—found no significant deviation between TPS and the guarded hot plate method, reinforcing the validity of the chosen approach.

## 3. Results and Discussion

The bulk density results are summarised in Table 1, with each entry presenting the mean value followed by the standard deviation calculated from five measurements. The data highlight notable variations in uncompressed bulk density based on the supplier and material composition. Supplier A’s fibre exhibits the lowest bulk density, measured at 15.59 ± 2.32 kg/m^3^. In contrast, the bulk density of the material provided by Supplier C, which includes a fibre and hurd mixture, is significantly higher, reaching 31.7 ± 2.43 kg/m^3^. This is nearly a twofold increase compared to Supplier A’s fibre and can likely be attributed to differences in the fibre diameter and the inclusion of hurd, which is known to possess a much higher bulk density than fibre alone. This is further supported by the bulk density measurements of the fine and coarse hurd from Supplier C, which are 106.48 ± 1.38 kg/m^3^ and 122.19 ± 0.97 kg/m^3^, respectively. These values far exceed the densities of fibre samples from all suppliers, underscoring the contribution of hurd to the uncompressed bulk density.

**Figure 3:**
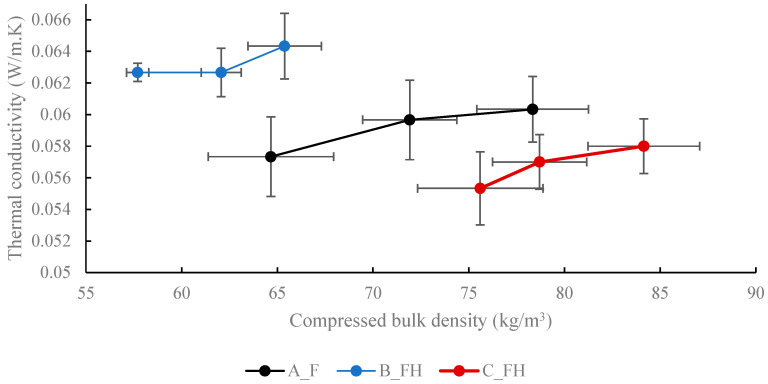
Thermal conductivity plotted against compressed bulk density.

The results of the thermal performance measurements (TPMs) are presented in Figure 3, where thermal conductivity is plotted against the compressed bulk density. Each marker represents the average of five measurements, with error bars indicating the standard deviations for both bulk density and thermal conductivity.

The thermal conductivity values ranged from a minimum average of 0.055 W/mK to 0.064 W/mK. The bulk density values under compression differ from the uncompressed measurements in Table 1, with each data point corresponding to compression loads of 1, 3, and 5 kg, respectively. As the applied weight increased, the bulk density rose significantly, particularly for the A_F samples, which lacked the less compressible hurd particles. Distinct differences were observed between suppliers, highlighting the influence of material composition and structure. Within each sample group, increasing the bulk density under compression resulted in a higher thermal conductivity. A similar trend was observed in a previous study on hemp shives [20]. This trend is likely due to a reduction in thermal contact resistance, both between the test material and the TPS sensor and among individual fibres and hurd particles. However, an inverse trend was noted for the different suppliers. For example, fibre from Supplier C, which exhibited the highest compressed bulk density, showed the lowest thermal conductivity. This discrepancy is likely attributable to variations in sample composition and fibre and hurd geometry, which can alter the material’s thermal performance. Our study found consistency with previous studies’ outcomes that indicate a decrease in thermal conductivity (becoming more insulating) with increasing density [5,6]. However, we found this only to be the case between the three different sample groups as indicated in Figure 3, and not the case with increasing the density within each individual sample group by increasing the compressive load. Our study showed higher readings of thermal conductivity (less insulating) compared with previous studies.

**Figure 4:**
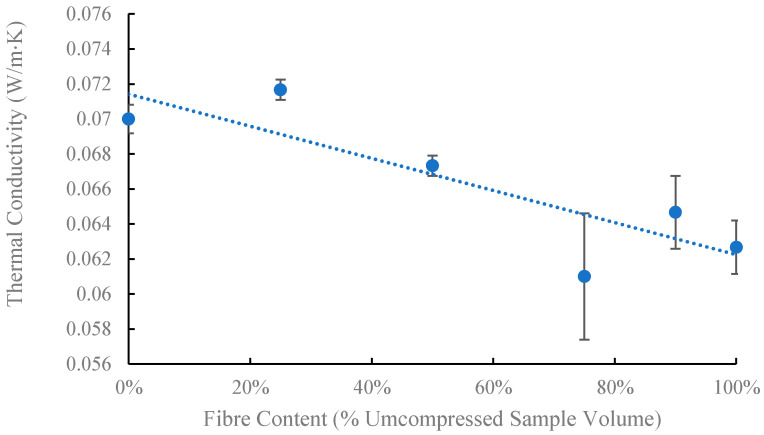
Thermal conductivity plotted against fibre content (blue dot indicates averaged values, and the dashed line is a linear regression of data points).

Figure 4 shows the influence of the fibre-to-hurd ratio on the thermal conductivity of compressed (1 kg) bulk mixtures, with six samples (Supplier B) varying in fibre content from 0% (hurd only) to 100% (fibre only). The results reveal a clear trend: increasing fibre content generally reduces thermal conductivity, suggesting improved thermal insulation properties. For instance, the sample with 100% fibre content exhibits the lowest thermal conductivity at 0.0627 W/mK, while the hurd-only sample (0%) shows a distinctly higher value of 0.070 W/mK. Interestingly, the normalised standard deviation of thermal conductivity measurements increases with higher fibre content, indicating greater variability in these samples. This may reflect challenges in achieving uniform fibre–hurd distribution in high-content mixtures. Nevertheless, the overall results support the conclusion that mixtures with high fibre content are advantageous for thermal insulation applications due to their lower thermal conductivities.

**Figure 5:**
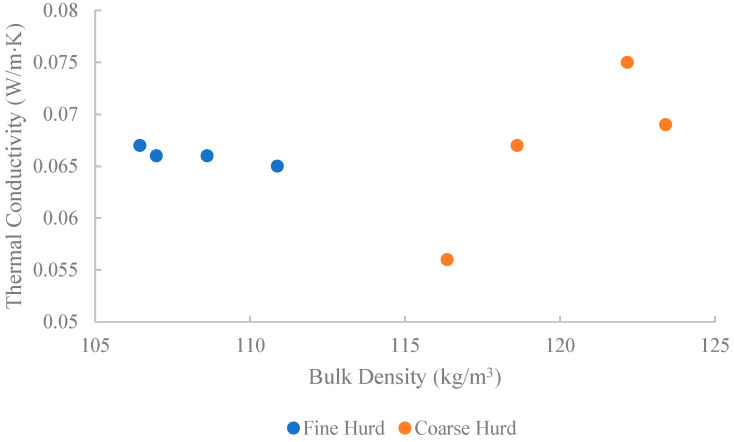
Thermal conductivity of bulk hurd samples.

The thermal conductivity of the compressed (1 kg) hurd-only samples was assessed using fine and coarse hurd particles from Supplier C, which were separated based on size to evaluate their impact on thermal properties. The results, shown in Figure 5, indicate similar average thermal conductivities for coarse hurd (0.067 W/mK) and fine hurd (0.066 W/mK), despite notable differences in bulk density. This conductivity is slightly lower compared to the hurd-only samples from Supplier A (0.070 W/mK, see Figure 4). Coarse hurd exhibited a higher average bulk density (122.19 kg/m^3^) compared to fine hurd (106.48 kg/m^3^). A key observation is the higher variability in thermal conductivity measurements for coarse hurd particles, with a standard deviation of 11.9% compared to only 1.2% for fine hurd. This increased variation is likely attributed to fewer hurd particles making contact with the TPS2 sensor surface, leading to a reduced representative measurement area and greater scattering of results. Overall, the thermal conductivity of hurd clearly exceeds that of hemp fibres.

To contextualise the thermal performance of hemp materials, comparative measurements were conducted using conventional reference insulation materials. Glass wool exhibited a thermal conductivity of 0.032 ± 0.0017 W/mK, while polystyrene was slightly higher at 0.035 ± 0.0043 W/mK. In comparison, the thermal conductivity of hemp-based samples was approximately twice as high. However, the same insulation properties (R-values) can be achieved by increasing the thickness of the hemp-based insulation layers, making them a viable alternative in applications where space constraints are less critical and an optimised carbon footprint is targeted.

Future research will focus on optimising the thermal insulation properties of hemp-based materials. Variations in thermal conductivity observed among different hemp suppliers suggest that differences in hurd-to-fibre ratios significantly influence performance. Additionally, other key factors, such as fibre length, fibre diameter, particle size distribution, and controlled variations in moisture content, will be systematically investigated using micro-computed tomography. These studies aim to enhance the understanding of thermal behaviour and drive further improvements in the design and application of hemp-based insulation materials.

## 4. Conclusions

This study demonstrates that unmanufactured hemp-based materials, specifically mixtures of hemp fibre and hurd, exhibit thermal conductivity values ranging from 0.055 to 0.065 W/mK. These values place them within a viable range for building insulation applications, particularly in sustainable construction where natural materials are prioritised.

Comparing the thermal performance of samples from different suppliers revealed distinct differences in hemp composition, bulk density, and thermal conductivity, highlighting the role of raw material variability in insulation performance. Notably, increasing compressive load and compaction led to higher bulk density and, consequently, increased thermal conductivity. This suggests that while compression may improve structural stability, it can reduce the material’s insulating capacity by facilitating heat transfer.

A key finding of this study is the significant influence of material composition on thermal properties. Higher fibre content consistently resulted in lower thermal conductivity, indicating that fibre-rich mixtures are preferable for enhanced insulation performance. Conversely, the presence of hurd, which possesses a higher bulk density, was associated with increased thermal conductivity. This underscores the importance of optimising the fibre-to-hurd ratio to balance insulation effectiveness and material stability.

While hemp-based insulation exhibits higher thermal conductivity compared to conventional materials such as glass wool or polystyrene, equivalent thermal resistance (R-values) can be achieved through increased insulation thickness. This makes hemp-based insulation a viable alternative in applications where space constraints are less critical and a reduced carbon footprint is a priority. Given the carbon sequestration potential of hemp during cultivation, its use as insulation material has the added benefit of offsetting embodied carbon in buildings.

Future research should focus on refining the material composition and processing methods to further optimise the thermal performance of hemp-based insulation. Key areas for investigation include the effects of fibre diameter, particle size distribution, and moisture content, as well as the potential benefits of incorporating binders to improve insulation efficiency. By enhancing the understanding of hemp’s thermal behaviour, this research contributes to the development of low-carbon insulation solutions that support sustainable building practices.

## Figures and Tables

**Table 1:** Results of the bulk density measurements.

ID	Supplier	Composition	Uncompressed Bulk Density [kg/m^3^]	Results
A_F	A	Fibre	15.59 ± 2.32	Figure 3
B_FH	B	Fibre and hurd	24.65 ± 2.86	Figure 3 and Figure 4
C_FH	C	Fibre and hurd	31.7 ± 2.43	Figure 3
C_H1	C	Fine hurd	106.48 ± 1.38	Figure 5
C_H2	C	Coarse hurd	122.19 ± 0.97	Figure 5

## Data Availability

Data will be made available upon request.
